# Chronic alcohol consumption disrupts the gut microbial and metabolic landscapes

**DOI:** 10.3389/fmicb.2026.1794794

**Published:** 2026-05-13

**Authors:** Madison B. Blanton, Ethan G. Napier, Katelyn E. Keen, Ethan V. Stuart, Isaac R. Cinco, Hami Hemati, Ronald C. Bruntz, Landon Wilson, Stephen Barnes, Rupak Khadka, Kathleen A. Grant, Ilhem Messaoudi

**Affiliations:** 1 Pharmaceutical Sciences, College of Pharmacy, University of Kentucky, Lexington, KY, United States; 2 Department of Microbiology, Immunology, and Molecular Genetics, College of Medicine, University of Kentucky, Lexington, KY, United States; 3 Molecular and Cellular Biochemistry, College of Medicine, University of Kentucky, Lexington, KY, United States; 4 Targeted Metabolomics and Proteomics Laboratory, University of Alabama at Birmingham, Birmingham, AL, United States; 5 Division of Pulmonary, Allergy, and Critical Care Medicine, Department of Medicine, University of Alabama at Birmingham, Birmingham, AL, United States; 6 Division of Neuroscience, Oregon National Primate Research Center, Oregon Health and Science University, Beaverton, OR, United States

**Keywords:** 16S rRNA sequencing, chronic alcohol consumption, macaque, metabolome, microbiome, monocytes, trained immunity

## Abstract

**Introduction:**

Alcohol use disorder (AUD) increases incidence of infections, organ damage, and cancers. Aberrant inflammation is likely a driver of these adverse outcomes. Indeed, chronic alcohol consumption (CAC) rewires macrophages/monocytes toward a hyper-inflammatory phenotype. Prior studies showed increased gut permeability and dysbiosis. Translocation of host- and microbial-derived metabolites could trigger the hyper-inflammatory responses generated by macrophages/monocytes. However, the exact changes in these metabolites remain poorly defined due to confounders that complicate clinical studies and the differences between human and rodent gut microbiomes.

**Methods:**

Here, we utilized a non-human primate model of ethanol self-administration to characterize alcohol-induced alterations in gut microbes and associated metabolomes. The microbiome was analyzed with 16s rRNA sequencing while a combination of GC-MS and LC-MS was used to assess changes in metabolites. Monocyte function was determined using flow cytometry.

**Results:**

Twelve months of alcohol use led to a decrease in SCFA-producing bacteria and disruption of fatty acid and amino acid metabolites. Moreover, fecal metabolites obtained after 12 months of CAC heightened monocytes' inflammatory responses.

**Discussion:**

These findings indicate that CAC-induced gut dysbiosis contributes to changes in fecal and circulating metabolites, which in turn can lead to monocyte dysregulation, possibly via innate immune training-like mechanisms.

## Introduction

The microbiome is an intricate network of bacteria, viruses, and fungi that modulates host health by influencing nutritional status, neurological state, and local and systemic immune system responses (Afzaal et al., 2022; Wiertsema et al., 2021). Consequently, gut dysbiosis has been associated with various immunological conditions such as asthma, chronic obstructive pulmonary disease (COPD), inflammatory bowel diseases, multiple sclerosis, type 1 diabetes, and rheumatoid arthritis (Dang and Marsland, 2019; Mousa et al., 2022). This delicate microbial ecosystem is highly susceptible to disruption by various environmental, dietary, and lifestyle factors, including alcohol misuse. Approximately 84% of adults report consuming alcohol, with an estimated 11% suffering from alcohol use disorder (AUD) (NIAAA, 2026).

Several cross-sectional studies have shown alcohol-induced disruptions in the composition of gut microbial communities. Common changes in the gut microbiome among patients with AUD and rodents after alcohol exposure include a decrease in the bacterial phyla Firmicutes, Bacteroidetes, and Verrucomicrobiota (Chen et al., 2022). Firmicutes, including short-chain fatty acid (SCFA) producers *Roseburia* and *Faecalibacterium*, play a role in fortifying tight junctions by promoting the expression of ZO-1 and occludins and the assembly of tight junctions (Tan et al., 2014). *Akkermansia*, from the phylum Verrucomicrobiota, facilitates intestinal integrity by promoting mucus production and helping to establish the gut mucosal layer (Grander et al., 2018). Given the role of these bacteria in maintaining gut barrier integrity, reduced abundance could lead to barrier hyperpermeability (Harberts and Schnabl, 2026). Impaired barrier function would allow microbial components, such as endotoxins and metabolites, to translocate into circulation. Indeed, we and others have shown an increase in the levels of circulating endotoxins in humans, non-human primates, and rodents after chronic alcohol consumption (CAC) (Barr et al., 2018; Lewis et al., 2021; Liangpunsakul et al., 2017; Nanji et al., 1993; Zhou and Zhong, 2017). Alcohol-induced changes in the circulating metabolome, such as branched-chain amino acids, cAMP and TCA signaling metabolites, and cortisol metabolites have also been noted (Cao et al., 2025; Freeman et al., 2011; Li et al., 2023; Zhao et al., 2021). Alterations in the levels of these circulating products have been shown to influence the function of peripheral immune cells and cause inflammation (Blomkalns et al., 2011; Kolypetri and Weiner, 2023; Calder, 2006; Yeager et al., 2008).

Interestingly, prolonged alcohol misuse results in skewing of monocytes toward a hyperinflammatory phenotype (Malherbe and Messaoudi, 2022; Sureshchandra et al., 2016). This exacerbated response could result from “innate immune training” (Netea et al., 2011), a concept that innate immune cells generate a heightened response upon subsequent exposure to a stimulus, resulting from metabolic and epigenetic modifications induced after initial low dose exposure or “training” (Netea et al., 2020). Although this reprogramming of innate immune cells could lead to enhanced protection against various pathogens, more recent work has described the potential for maladaptive immune training (Bekkering et al., 2021, 2014). Therefore, it is possible that alcohol-mediated hyperpermeability of the intestinal barrier can result in the translocation of endotoxins and other microbial products into circulation, which may subsequently lead to the maladaptive “training” of circulating monocytes, causing hyperinflammatory responses. However, the link between the two remains unclear.

Many of the findings in alcohol-related research have been collected from cross-sectional clinical studies or rodent models, and while these studies have contributed significantly to our current understanding of the impact of CAC on the gut barrier, they have inherent limitations. Longitudinal data on gut microbial or microbiome alterations before and following alcohol consumption are limited in humans. In fact, most clinical studies are conducted using samples from patients with AUD and liver disease. Additionally, the microbial communities of inbred and specific-pathogen-free (SPF) rodent models differ significantly from those of humans. Rhesus macaques, on the other hand, share significant genetic and physiological similarities with humans (Chiou et al., 2020). Moreover, the macaque gut microbiome is closely related to that of humans (Clayton et al., 2016). Despite these advantages, only a few non-human primate studies have investigated the impact of chronic alcohol use on the microbiome or metabolome (Barr et al., 2018; Piacentino et al., 2021; Zhang et al., 2019).

In this study, we leveraged access to plasma and fecal samples from a model of continuous voluntary ethanol self-administration where rhesus macaques engaged in 12 months of unrestricted daily drinking. Our study demonstrated that prolonged alcohol consumption reduces levels of SCFA, likely a result of reduced abundance of several commensals that are known for producing SCFA and promoting mucus secretion. Moreover, fecal metabolites led to enhanced monocyte responses. These findings improve our understanding of the role of gut microbiota in the progression of immune-related diseases following alcohol consumption.

## Methods

### Ethics declaration

All animal work was approved by Oregon Health and Science University (OHSU) West Campus Institutional Animal Care and Use Committee (IACUC). The macaques sampled for this study were handled in accordance with ONPRC animal care program, fully accredited by AAALAC International and is based on the laws, regulations, and guidelines set forth by the United States Department of Agriculture [e.g., the Animal Welfare Act and Animal Welfare Regulations, the Guide for the Care and Use of Laboratory Animals, 8th edition (Institute for Laboratory Animal Research), and the Public Health Service Policy on Humane Care and Use of Laboratory Animals]. At the conclusion of the study, ketamine was used for sedation to transport macaques to the necropsy suite. Once secured, sodium pentobarbital was administered intravenously, and the macaques were assessed for deep and steady respirations as well as a lack of palpebral, corneal, and deep pain withdrawal reflexes before the harvest of any tissues (Davenport et al., 2014; Daunais et al., 2014). Additionally, all methods are reported in accordance with ARRIVE guidelines.

### Cohort description and sample collection

Samples were collected through the Monkey Alcohol Tissue Research Resources (MATRR; www.matrr.com). The MATRR is a repository of tissues collected from a non-human primate model of voluntary ethanol self-administration. Samples from a total of 63 animals were utilized throughout the duration of this study, and individual demographics of the rhesus macaques were from MATRR cohorts 5, 6a, 14, 16, 17, 20, and 22. Briefly, rhesus macaques are trained to utilize operant drinking panels and then undergo schedule-induced polydipsia for 4 months with 16-h drinking sessions, increasing in dose every 30 days (Grant et al., 2008). Macaques then have “open access” and can choose between drinking water and a 4% ethanol w/v solution for 22 h/day throughout the remainder of the study, and their drinking category is determined as previously described (Baker et al., 2023, 2014; Grant et al., 2008). All animals are housed in individual quadrant cages maintained at a constant temperature and subjected to an 11-h light cycle. For sampling, macaques remained in their home cages and were trained to present their legs for blood collection while fecal samples were obtained from fresh-catch stool collection. Plasma and feces were stored at −80 °C while PBMC were cryopreserved until use.

### 16s amplicon sequencing and bioinformatics analysis

Amplification of the hypervariable V4 region of the 16s rRNA gene was performed using the 515F/806R PCR primers and analyzed as previously described (Cinco et al., 2024). The forward primers were conjugated with a 12-bp barcode (Parada et al., 2016). Each reaction was run in duplicate and prepared with GoTaq master mix (Promega Corporation, Madison, WI). Cycling conditions were as follows: 94 °C for 3 min, 37 cycles of 94 °C for 45 s, 50 °C for 1 min, and 72 °C for 1 min, followed by a final cycle at 72 °C for 10 min. The PCR products were multiplexed using Quant-iT PicoGreen dsDNA Assay Kits and dsDNA Reagents (ThermoFisher Scientific, Waltham, MA). The resulting library was spiked with ~15%−20% PhiX and sequenced on an Illumina MiSeq. Raw FASTQ 16s rRNA gene amplicon sequences were processed using the QIIME2 analysis pipeline (Bolyen et al., 2019). Sequences were demultiplexed and filtered using DADA2 (Callahan et al., 2016). MAFFT was used to align the sequence variants while FastTree2 was utilized to construct a phylogenetic tree (Katoh and Standley, 2013; Price et al., 2010). Taxonomy was assigned to sequence variants using q2-feature-classifier against the SILVA database (release 138) (Quast et al., 2013). The samples were rarified to 77,839 sequences per sample. QIIME2 was also used to generate alpha diversity metrics, while beta diversity was estimated using weighted and unweighted UniFrac distances (Lozupone et al., 2011). Differentially abundant bacteria between groups were identified via the LEfSe algorithm with a linear discriminant analysis score cutoff of 2 (Segata et al., 2011). PICRUSt2 was used to infer metagenomic and functional differences between groups, and MAASLIN2 was leveraged for regression analysis.

### Sample preparation for fecal metabolomics

Feces (~100 mg) were freeze-dried and the resulting fecal powders (10 mg) were treated with ice-cold 80% aqueous methanol for 30 min to extract metabolites. The extracts were centrifuged at 1,750*g* for 10 min at 4 °C, and the supernatants were transferred to a fresh glass tube and dried to completion under N_2_. The dried samples were resuspended in 0.1% formic acid in ddH_2_O (1 mL) before mass spectrometry analyses.

### Sample preparation for plasma metabolomics

A 100 μL aliquot of plasma specimen was placed in a 96-well Phenomenex Phree Phospholipid Removal Plate (Torrance, CA). For each well, acetonitrile (600 μL) containing 1% formic acid was added to each plasma specimen. Samples were then aspirated several times to allow thorough mixing to aid in protein precipitation on the plates. The 96-well plates were then vortexed for 10 min and eluted on a Phenomenex Presston 1000 positive pressure vacuum manifold at 10 psi for 1 min and collected in a 96-deep well collection plate (Torrance, CA). The eluted samples were then dried thoroughly under nitrogen gas using a Biotage SPE Dry 96 unit at 40 °C (Charlotte, NC). Samples were resuspended in 0.1% formic acid (200 μL). A pooled quality control sample containing an equal volume portion of each specimen was generated to evaluate the chromatographic and mass spectrometry consistency throughout the analysis.

### Analysis for fecal and plasma metabolomics

For each specimen analyzed, an aliquot (5 μL) of each sample was loaded onto a Phenomenex 2.1 x 100 mm, 1.6 μm Luna Omega, 80 Å reverse-phase column (Torrance, CA). A linear gradient of 2%−50% mobile phase B for 5 min, then 50%−98% B until 6.0 min with a 1-min hold, then re-equilibration at initial conditions for 3 min using an Exion UHPLC (Sciex, Toronto, Ontario) and a flow rate of 500 μL/min. The mobile phases are (A) ddH_2_O with 0.1% formic acid and (B) acetonitrile with 0.1% formic acid, respectively. A SCIEX 7600+ ZenoTOF mass spectrometer (SCIEX, Toronto, Canada) was used to analyze the metabolite profile. The IonSpray voltages for positive and negative modes were +/– 5,500/4,500 V and the declustering potential was +/– 40 V. IonSpray GS1/GS2 and curtain gases were set at 50 psi. The interface heater temperature was 500 °C. In each duty cycle (250 msec), eluted compounds were subjected to a time-of-flight survey scans (100 msec) from *m/z* 50–1,000. Product ion time-of-flight scans to obtain the tandem mass spectra of the selected molecular ion(s) over the range from *m/z* 50–1,000 were collected over 10 ms intervals using a collision energy spread of 5 eV with a set collision point of 20 eV. A ZenoTrap triggering threshold of 20,000 cps was used to enhance lower-abundant species product ion spectra. Spectra were centroided and de-isotoped by Analyst OS software, version 3.4 (SCIEX, Toronto, Canada).

LC-MS data were processed using MS-Dial 5.4 (RIKEN Center, Yokohama City, Kanagawa, Japan) and identification of metabolites was assessed against the IROA 600 standard compound library (IROA Technologies, Sea Girt, NJ) and verified by evaluating product ion spectra of each target using PeakView 2.2 software (SCIEX, Toronto, Ontario). Statistical analysis was completed using MetaboAnalyst 6.0 (https://www.metaboanalyst.ca/). Given the large diversity of the fecal metabolome and our small cohort size, fecal metabolites with a log(2) fold change threshold of ±0.585 and a nominal *p*-value threshold of ≤ 0.05 were considered differentially abundant. In contrast, as plasma contains a less diverse range of metabolites, plasma metabolites with a log(2) fold change threshold of ±0.585 and an FDR-corrected *p*-value threshold of ≤ 0.05 were considered differentially abundant. A *p*-value cutoff of < 0.1 and a minimum of two hits were required for pathway enrichments of metabolomics data.

### Short-chain fatty acid GC/MS sample preparation and analysis

Fatty acids were extracted by adding 500 μL water with 5 mg *d*_7_-butyric acid to frozen fecal powder followed by concentrated HCl (100 μL) and methyl tert-butyl ether (MTBE; 100 μL). The slurry was vigorously homogenized for 3 min then incubated for 30 min at room temperature. Samples were centrifuged and the upper MTBE layer was transferred to tubes containing Na_2_SO_4_ and incubated for 10 min at room temperature. Samples were centrifuged at 14,000*g* for 1 min and supernatant (20 μL) was added to low-recovery glass insert along with MTBSTFA (5 mL; Restek, Bellefonte PA, Cat #: 35608) and derivatized at 80 °C for 30 min. Derivatized samples were run on an Agilent 5977B GC/MS containing an Agilent HP-5MS column. The inlet was set at 250 °C with a pressure of 4.9 psi and septum purge flow of 3 mL/min. The split inlet mode ratio was set to 10:1 and split flow of 7.67 mL/min. Initial oven temperature was set to 50 °C for 1 min, followed by a linear ramp to 300 °C at a rate of 11.5 °C per minute. Full-scan MS detection range was set to 50–550 *m/z* with a source and quad temperature of 230 °C and 150 °C, respectively. Data files were imported into MassHunter Qualitative Analysis (Agilent) for peak and mass spectrum extraction. Acetate, propionate, isobutyrate, *n*-butyrate, 2-methylbutyrate, isovalerate, and *n*-valerate were confirmed using Short-chain Fatty Acid Mixture 2 (Cayman, Ann Arbor MI, Cat #: 28680).

### Sample preparation for monocyte metabolomics

Monocytes were sorted from cryopreserved total PBMC using EasySep™ APC Positive Selection Kit II (Stemcell), and ~150,000 monocytes/well were added to a round-bottom 96-well plate. Monocytes were stimulated with 0.5 μg/mL LPS in RP10 or RP10 only (500 mL RPMI, 50 mL FBS, 5 mL Pen/Strep, 5 mL l-glutamine) for 4 h in a 37 °C, CO_2_ incubator. Following stimulation, monocytes were washed twice with 0.9% NaCl and ~100,000 cells per sample were resuspended in cold methanol and isopropyl myristate and stored at −80 °C until use. Monocytes were then extracted with 50% MeOH. Following addition of MeOH, mixtures were placed on ice for 20 min and briefly vortexed at 5 min intervals. Following centrifugation at 24,000*g* for 10 min at 4 °C, the aqueous phase containing polar metabolites was filtered to remove lipids using Agilent captiva EMR-Lipid filter under positive pressure manifold. The resulting filtrate was fully dried at 10^−3^ mBar using a SpeedVac (Thermo Fisher Scientific, Waltham, MA, USA) to evaporate remaining MeOH; then reconstituted in 5% acetonitrile: LCMS grade water.

### Analysis of monocyte metabolomics

Since metabolite levels are lower in monocytes, we used a targeted method on a triple quadrupole mass spectrometer to improve sensitivity. Metabolite separation was achieved using the Agilent 1290 Infinity II Stainless HPLC system equipped with an InfinityLab Poroshell 120 HILIC-Z column. The mobile phase consisted of solvent A (water with 10 mM ammonium acetate and 0.1% medronic acid) and solvent B (acetonitrile with 10 mM ammonium acetate). The flow rate was set at 0.400 mL/min, and the gradient elution program was as follows: 90% B at 0.00 and 1.00 min; decreasing to 78% B at 8.00 min, 60% B at 12.00 min, and 10% B at 15.00 min; held at 10% B until 18.00 min; then returned to 90% B at 19.00 min and held until 23.00 min. Analysis performed on Agilent 6495C LC/TQ with the following parameters: ion mode set at both positive and negative, gas temperature 200 °C, drying gas flow 14 L/min, nebulizer gas at 50 psi, sheath gas temperature at 375 °C, sheath gas flow at 12 L/min, capillary voltage set to (+)3,000/(–)2,500 V and nozzle voltage set at 0 V.

After data acquisition, initial data analysis performed on Agilent MassHunter Quantitative Analysis for peak identification and quantification. Relative abundance of each metabolite was then exported into Excel and statistical analysis performed using Metaboanalyst (https://www.metaboanalyst.ca). Graphs were made using GraphPad Prism.

### Monocyte stimulation with fecal metabolites

Briefly, control monocytes were sorted from cryopreserved total PBMC using EasySep™ APC Positive Selection Kit II (Stemcell), and ~150,000 monocytes/well were added to a round-bottom 96-well plate. Monocytes were stimulated with fecal metabolites at a 1:4 dilution with RP10 or RP10 only for 24 h in a 37 °C, CO_2_ incubator. Monocytes were then washed with RP10 and replated in a 96-well flat-bottom plate. Monocytes were allowed to rest for 120 h (5 days) in a 37 °C, CO_2_ incubator. After 72 h of rest (day 3), media was replenished by removing 100 μL of supernatant and adding 100 μL of pre-warmed RP10. After the resting period, monocytes were stimulated with 0.5 μg of LPS in RP10 for 6 h. After 1 h of stimulation, Brefeldin A (Sigma, St. Louis MO) was added. Cells were surface-stained with CD14 (APC), CD16 (PB), and HLA-DR (APC-Cy7), fixed, permeabilized, and then stained for intracellular TNFα (Per-CP) and IL-6 (PE). Stained samples were then acquired on the Attune NxT Cytometer (ThermoFisher Scientific) and analyzed using FlowJo (BD).

### Statistical analysis for SCFA and trained immunity experiment

Statistical analysis was performed using GraphPad Prism software (GraphPad Software Inc., La Jolla, CA). Outliers were identified via the ROUT test (*Q* = 1%). Normality of data was determined via the Shaprio–Wilk test. If the data passed the normality test (α = 0.05), a two-tailed unpaired *T*-test with Welch's correction was applied. If the data did not pass the normality test, comparisons were carried out using a two-tailed, Mann–Whitney test.

## Results

### Daily alcohol consumption for 12 months dysregulates gut microbial communities

**Figure 1:**
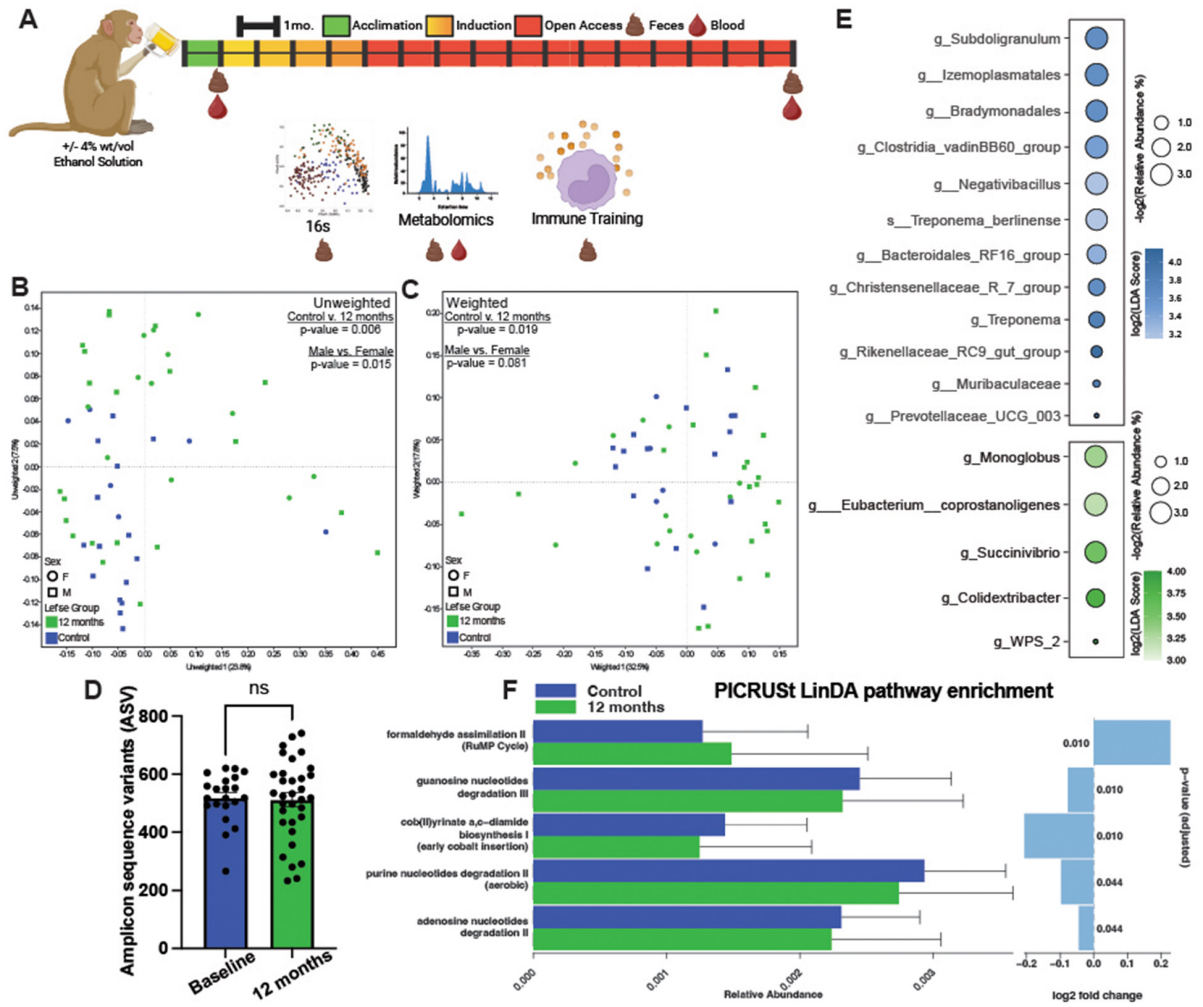
Alcohol misuse results in the loss of commensal and SCFA-producing bacteria. **(A)** Study design. **(B)** Unweighted and **(C)** weighted PCoA visualization of fecal samples collected at baseline and after 12 months of daily ethanol consumption. **(D)** Bar plot depicting the amplicon sequence variants (ASVs) identified in fecal samples collected at baseline and after 12 months of daily ethanol consumption. Error bars were defined as ±standard error of the mean (SEM). **(E)** Bubble plot of differentially abundant bacteria in feces after 12 months of daily drinking compared to controls. The size of the bubble represents relative abundance and color denotes LDA score. **(F)** Bar graph of pathways PIECRUSt predicted to be altered based on 16S data. For the 16s rRNA sequencing experiments: *n* = 21 controls and *n* = 33 after 12 months of drinking.

We assessed alcohol-induced changes in the gut microbial community and the intestinal and peripheral metabolites via 16S rRNA sequencing and a combination of GC-MS and LC-MS, respectively. Subsequently, we evaluated how these changes contributed to the cytokine responses by monocytes (Figure 1A). The unweighted (presence or absence of species) and weighted (relative abundance of species) principal coordinate analysis (PCoA) of 16S RNA sequencing data showed a significant distinction between community profiles based on alcohol consumption, albeit only the unweighted analysis denoted a significant difference between sexes (*n* = 21 controls and *n* = 33 after 12 months of drinking; Figures 1B, C). Despite the differences in gut microbial composition, the overall number of unique microbial sequences remained unchanged with CAC, as measured by amplicon sequence variants (ASVs; Figure 1D).

Differentially abundant bacteria were identified using Linear Discriminant Effect Size (LEfSe) analysis after 12 months of daily drinking compared to baseline. The relative abundance of SCFA producers (*Prevotellacae, Muribaculacae, Rikenellaceae, Christensenellaceae, Bacteroidales, Clostridia)* was decreased after 12 months of drinking compared to controls (Figure 1E). Interestingly, we observed an increase in the relative abundance of bacteria that participate in the fermentation of carbohydrates and ultimately could contribute to the production of SCFA (*Colidextribacter, Succinivibrio, Eubacterium, Monoglobus*). We also performed MaAsLin2 analysis to correlate average blood ethanol concentration (BEC) with bacterial abundance (Supplementary Figure 1). This analysis revealed that the relative abundance of the genus *Subdologranulum* was positively correlated to increased ethanol consumption. The *Anaerosporobacter* genus, Selenomonadaceae family, *Fibrobacter* genus, and *Bradymonadales* genus were negatively correlated with BEC. Abundance of *Bradymonadales* was also significantly decreased after 12 months of daily drinking, which may indicate this microbe is an indicator of chronic alcohol use (Figure 1E, Supplementary Figure 1).

Next, we used phylogenetic investigation of communities by reconstruction of unobserved states (PICRUSt), a workflow that uses 16s amplicon sequencing data, to infer the functional capabilities of the microbiome (Langille et al., 2013). We focused on pathways that were present in >10% of samples and had an abundance of >0.1% (Figure 1F). Five metabolic pathways were predicted to be altered after alcohol consumption when using linear models for differential abundance analysis (LinDA; Figure 1F), including an increase in formaldehyde assimilation and a concurrent decrease guanosine, purine, and adenosine degradation. Together, these data suggest that 12 months of daily alcohol consumption dysregulates the abundance and diversity of SCFA producers in the gut and could have implications for nucleotide metabolism.

### Prolonged alcohol use alters fatty acid and amino acid metabolism

We then performed untargeted metabolomics with LC-MS on the feces to assess the metabolic changes after 12 months of alcohol consumption (*n* = 9 controls and *n* = 8 after 12 months of drinking; Supplementary Table 1). A sparse partial least squares discriminant analysis (sPLSDA) showed distinct separation of the fecal metabolome before and after 12 months of daily drinking (Supplementary Figure 2A). Given the large diversity of the fecal metabolome and our small cohort size, fecal metabolites with a log(2) fold change threshold of ±0.585 and a nominal p-value threshold of ≤ 0.05 were considered differentially abundant. Chronic ethanol consumption for 12 months led to increased abundance of lithocholic acid (LCA), proline, quinolinate, glucose 1-phosphate (G1P), and L-carnitine, and reduced abundance of thiamine monophosphate (TMP), xanthosine monophosphate (XMP), uridine monophosphate (UMP), and cytidine monophosphate (CMP; Supplementary Figure 2B). These changes were reflected in the pathway enrichment analysis with increased oxidation of fatty acids, whereas reduced XMP, UMP, and CMP indicate downregulation of purine and pyrimidine metabolism (Jurecka and Tylki-Szymanska, 2022) as previously reported in rodents following alcohol consumption (Zhang et al., 2017) (Supplementary Figure 2C). These results are also in line with our PICRUSt results showing downregulation of nucleotide degradation (Figure 1F).

**Figure 2:**
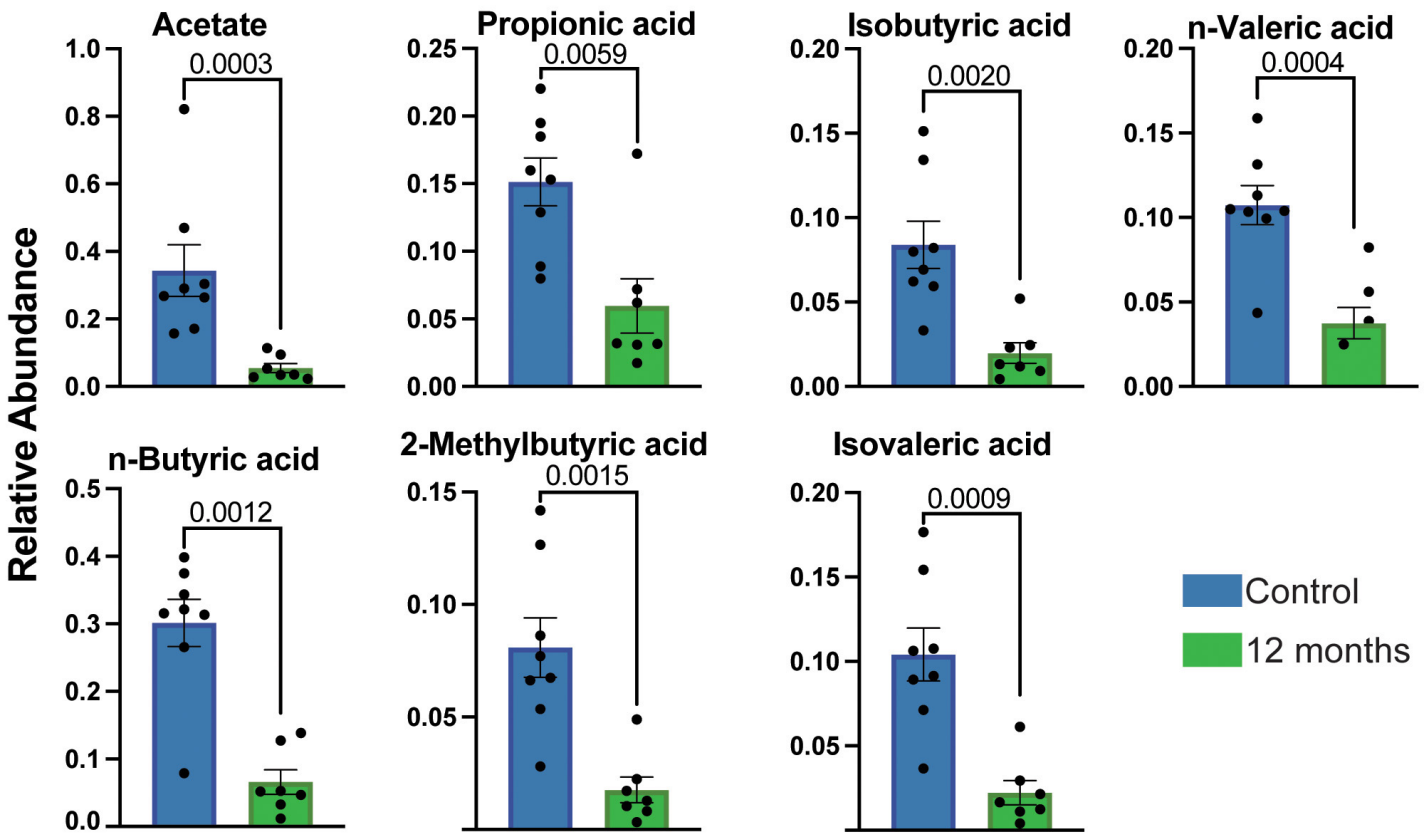
The overall abundance of short chain fatty acids is decreased in stool samples following CAC. Bar graphs showing the relative abundance of SCFA identified in stool samples before and after 12 months of open access to alcohol using targeted metabolomics (GC-MS). Error bars were defined as ±standard error of the mean (SEM). For the fecal GC-MS experiments: *n* = 8 controls and *n* = 7 samples after 12 months drinking.

To better understand the implications of changes in SCFA producers on the metabolome, we conducted targeted GC/MS analysis on fecal samples (*n* = 8 controls and *n* = 7 samples after 12 months drinking). In line with the loss of SCFA producers at 12 months of CAC, significant decreases in acetate, propionate, isobutyrate, *n*-butyrate, 2-methylbutyrate, isovalerate, and *n*-valerate were noted after 12 months of alcohol consumption (Figure 2). We were also able to identify changes in several medium and long-chain fatty acids. Specifically, we observed a significant decrease in palmitic, oleic, stearic, 11-eicosenoic, nonadecanoic, dodecanoic, and behenic, acids, while only modest changes were observed in the levels of 10-nonadecenoic and lignoceric acids (Supplementary Figure 3). Together, these data indicate that alcohol-mediated disruption of metabolism contributes to significant dysregulation of nucleotide and SCFA pathways.

**Figure 3:**
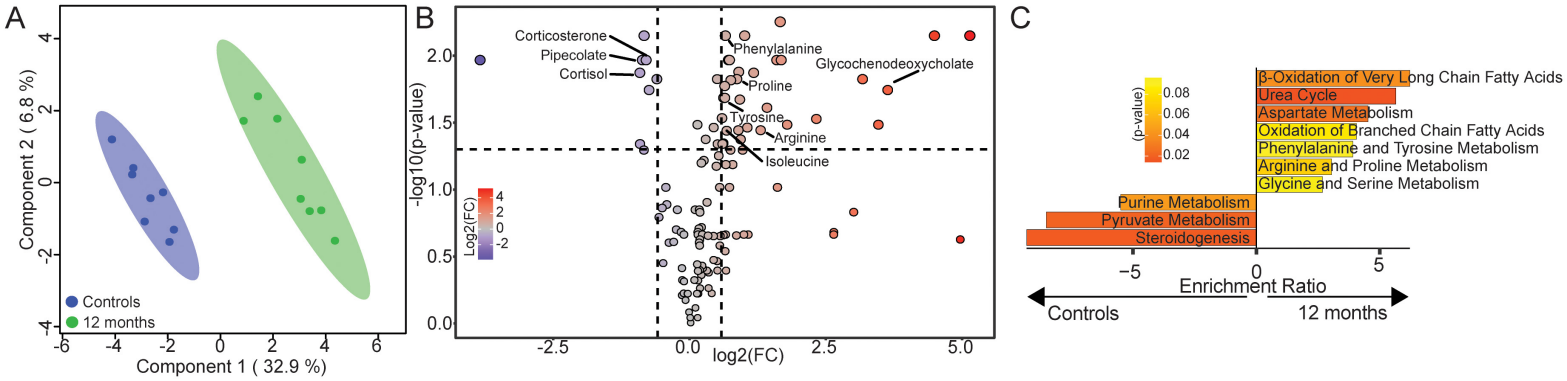
Twelve months of alcohol use induces a similar metabolic profile in the periphery to that observed in the gut. **(A)** sPLSDA of LC-MS analysis of macaque plasma samples from ethanol naive controls and after 12 months. **(B)** Volcano plot of differentially abundant metabolites in plasma samples after 12 months. **(C)** Bar plot depicting selected enrichments of differentially abundant metabolites in plasma samples after 12 months (terms with *p*-value < 0.1 and >2 genes). For the plasma LC-MS experiments: *n* = 8 controls and *n* = 8 samples after 12 months drinking.

To determine whether changes in fecal metabolites were reflected in the periphery, we performed untargeted LC-MS on plasma samples from control (*n* = 8) and drinking animals after 12 months of open access (*n* = 8; Figure 3, Supplementary Table 1). An sPLSDA analysis revealed distinct separation in the plasma metabolome between control and animals after 12 months of alcohol use (Figure 3A). These differences were driven in part by the elevated levels of glycochenodeoxycholate, isoleucine, phenylalanine, proline, tyrosine, and arginine, concurrent with a decrease in pipecolate, cortisol, and corticosterone (Figure 3B). These metabolites suggest an increase in fatty acid oxidation and select amino acid metabolism (aspartate, phenylalanine, tyrosine, arginine, proline, glycine, serine), with a concomitant decrease in steroidogenesis (Figure 3). Collectively, these findings indicate that 12 months of alcohol consumption dysregulates the circulating and fecal metabolomes that are involved in similar amino acid and fatty acid metabolic pathways.

### Polar fecal metabolites following alcohol consumption increased the inflammatory response of circulating monocytes

**Figure 4:**
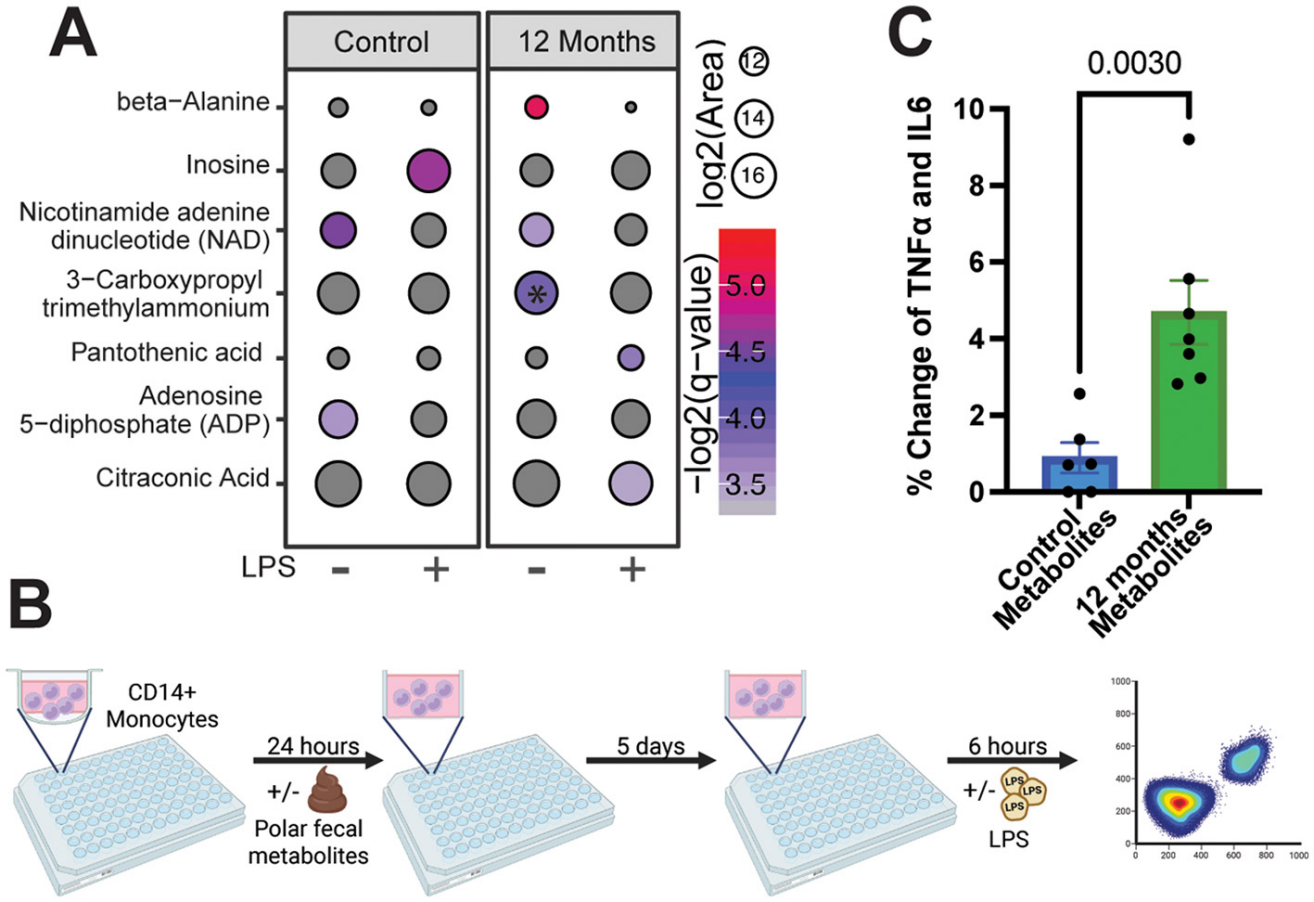
Monocytes are metabolically rewired following alcohol consumption, likely a result of immune training by fecal metabolites. **(A)** Bubble plot representing the changes in the monocyte metabolome in the presence or absence of LPS after 12 months of daily ethanol consumption. * Denotes significance when comparing the metabolite abundance between control and alcohol, while color denotes significance when compared to the non-stimulated/stimulated condition. Monocytes from *n* = 3 controls and *n* = 3 monocytes after 12 months of drinking were used. **(B)** Experimental design for trained immunity experiments. **(C)** Bar plot depicting the change in the percentage of cells producing TNFα, IL-6, or both (TNFα + IL6+) following LPS stimulation when compared to the non-stimulated response. Each dot represents an animal. For the trained immunity experiments, feces from *n* = 6 controls and *n* = 7 samples after 12 months drinking were used. Error bars were defined as ±standard error of the mean (SEM).

Chronic ethanol consumption skews monocytes toward a hyperinflammatory response (Lewis et al., 2021). Therefore, we examined the monocyte metabolome following 12 months of ethanol consumption (monocytes from *n* = 3 controls and *n* = 3 after 12 months), using untargeted LC-MS at rest and after LPS stimulation. We observed a higher abundance of 3-carboxypropyl trimethylammonium at rest in monocytes after 12 months of alcohol misuse compared to those of controls (Figure 4A). After stimulation, control monocytes showed a significant increase in inosine and a decrease in NAD and ADP abundance (Figure 4A). On the other hand, LPS-stimulated monocytes after 12 months of CAC showed a significant increase in the abundance of pantothenic and citraconic acid, concurrent with a decrease in beta-alanine, NAD, and 3-carboxypropyl trimethylammonium (Figure 4A). The increase of pantothenic acid could affect glycolysis as it is a precursor for coenzyme A, which is necessary for glycolytic flux and several other metabolic pathways (Marrocco and Ortiz, 2022). Increased citraconic acid, an isomer of itaconate, suggests disruption of the TCA cycle since cis-aconitate can be shunted toward itaconate via IRG1 (Chen et al., 2022).

To interrogate the interplay between ethanol induced changes in metabolites and response to LPS stimulation, monocytes from control animals were incubated with polar fecal metabolites isolated from macaques after 12 months of CAC (*n* = 7) or from controls (*n* = 6) followed by a short-term LPS stimulation (Figure 4B, Supplementary Figure 4). Monocytes exposed to fecal metabolites from animals after 12 months CAC generated larger responses to LPS compared to those exposed to metabolites from control animals, indicated by the increased frequency of monocytes producing TNFα and/or IL6 (TNFα, IL6+, or TNFα + IL6+; Figure 4C). Collectively, these findings indicate that shifts in microbiome metabolism following chronic alcohol misuse can enhance monocyte responses.

## Discussion

In this study, we assessed the changes in the fecal microbiome and their implications on gut and circulating metabolomes by leveraging access to fecal samples and plasma collected from a non-human primate model of continuous, unrestricted voluntary ethanol self-administration before and after 12 months of alcohol intake. While the number of unique amplicons was similar, gut microbial diversity and composition were significantly changed. We observed a significant loss of microbes capable of producing SCFA after 12 months of drinking. Moreover, the reduced Firmicutes and Bacteroidetes levels align with previous reports in rodent and humans following alcohol intake (Chen et al., 2022; Yan et al., 2011).

Metabolomics data from stool samples were in line with 16s results, indicating a significant reduction in SCFA abundance at 12 months. SCFAs, particularly butyrate, are important for maintaining gut integrity. Indeed, colonic epithelial cells predominantly use butyrate as energy to sustain cell-cell junctions (Barcenilla et al., 2000), and loss of butyrate producers impacts the gut lining (Hodgkinson et al., 2023). These data suggest that a loss of SCFAs and their bacterial producers following chronic alcohol use induces a metabolic shift in the gut, potentially impacting its permeability. Moreover, the significant loss of SCFA levels after 12 months of CAC was associated with an increase in metabolites associated with the β-oxidation of fatty acids, including l-carnitine. Elevated l-carnitine levels may result from the reduced abundance of Firmicutes, as they are responsible for converting l-carnitine into trimethylamine (TMA) (Vallance et al., 2018). The loss of SCFA-producing bacteria and SCFA levels might play a significant role in the clinical outcomes of AUD (Duan et al., 2023; Silva et al., 2020; Xiong et al., 2022). SCFAs decrease the production of inflammatory mediators such as IL6, TNFα, and IL1β, while increasing levels of anti-inflammatory mediators like IL10, TGF-β, and annexin A1. As a result, the loss of these metabolites could promote an inflammatory environment, impairing immunoregulatory functions and contributing to conditions such as liver disease, diabetes mellitus, depression, stress, and anxiety-like behaviors, which are associated with AUD (Duan et al., 2023; Silva et al., 2020; Xiong et al., 2022; Shukla and Hsu, 2025; Pohl et al., 2022; Zhang et al., 2021). Additionally, SCFAs influence blood lipid levels and blood pressure—highlighting their possible role in mediating the cardiovascular complications observed in AUD patients (Xiong et al., 2022; Kiechl et al., 1998). Overall, these findings suggest that SCFAs are a potential therapeutic target to mitigate alcohol-related organ damage.

Following 12 months of alcohol exposure, we measured increased levels of glucose-1-phosphate in feces, which may promote the Warburg effect in intestinal cells and facilitate the inflammatory phenotype associated with alcohol misuse (Bose et al., 2021; Palsson-Mcdermott and O'neill, 2013). Moreover, enhanced proline, LCA, and quinolinate, metabolites with immunomodulatory properties, were previously linked with CAC (Leclercq et al., 2021; Mrdjen et al., 2023; Nahar et al., 2022; Xu et al., 2023). We also observed a decreased abundance of TMP, probably due to impaired absorption of thiamine with CAC (Subramanya et al., 2010), and adenosine diphosphate ribose (ADP-ribose), a consequence of alcohol consumption-mediated reduction of NAD^+^ level (French, 2016).

The loss of SCFA producers and reduction in SCFA levels suggest a decrease in gut barrier function and an increased risk of gut leakage; therefore, we investigated the peripheral metabolome. We observed metabolic shifts associated with alcohol misuse, including changes in amino acid and lipid metabolism. These changes recapitulate trends noted in humans following alcohol misuse, bolstering the translational value of the NHP model. The circulating levels of isoleucine, corticosterone, cortisol, pipecolate; trends in purine and pyruvate metabolism, and steroidogenesis after 12 months of alcohol misuse in the macaque are in accordance with changes observed in human patients with a history of alcohol misuse (Badrick et al., 2008; Panicek et al., 1986; Richardson et al., 2008; Blaine et al., 2019; Zhang et al., 2017). It has been suggested that changes in circulating amino acid levels are secondary to dysregulated intestinal absorption (Butts et al., 2023; Dedon et al., 2025; Voutilainen and Kärkkäinen, 2019). Moreover, increased plasma metabolites associated with fatty acid oxidation and ketone bodies are likely byproducts of ethanol metabolism in the liver (Baraona and Lieber, 1979; Lu and George, 2024). We also observed elevated levels of circulating glycochenodeoxycholate (GCDC), which has been reported in patients with ALD (Hirata et al., 2024). We have previously reported that 12 months of CAC in our model system is not sufficient to induce elevated levels of circulating liver enzymes, AST and ALT (Lewis et al., 2021). However, since GCDC is hypothesized to be a predictive biomarker of liver injury, this suggests the animals are in the early stages of developing alcohol-related liver disease (Hirata et al., 2024). We also observed increased levels of isoleucine in the plasma after 12 months, a metabolite associated with enhanced PBMC activation (Zhenyukh et al., 2017), which could contribute to the hyper-inflammatory phenotype of monocytes observed after 12 months of alcohol consumption (Lewis et al., 2021).

Interestingly, we observed a high degree of overlap in the affected pathways in plasma and fecal samples. Both compartments reported enhanced levels of metabolites enriching to fatty acid oxidation pathways and tyrosine metabolism, concurrent with a reduction in purine metabolism, likely a result of an enhanced energy demand. We propose that the convergence between the two metabolome profiles could be a consequence of increased gut permeability and the translocation of small molecules from the intestines into circulation. In fact, we have shown increased levels of circulating endotoxin and a positive correlation between alcohol intake and endotoxin-core IgM antibody titers in this model, indicative of decreased barrier function (Barr et al., 2018; Lewis et al., 2021). Herein, we show monocytes treated with polar fecal metabolites isolated from stool samples collected from macaques after 12 months of CAC produced higher levels of IL-6 and TNF-α compared to monocytes treated with metabolites isolated from the stool of control macaques. These data provide preliminary evidence that components in the fecal metabolome associated with CAC have the potential to train monocytes. Future work should focus on elucidating what specific metabolites are driving this phenotype.

This study has some limitations. While we could assess sex as a biological variable (SABV) for alcohol-related changes in the microbiome, we were not statistically powered to examine SABV for metabolomic or trained immunity assays. Due to the small study size, comparisons were performed between macaques identified as very heavy/heavy drinkers vs. non-drinking macaques. We acknowledge that this approach limits our ability to study dose-dependent relationships. The current study's sample size also limits statistical power when assessing the impact of alcohol on the intestinal metabolome. Therefore, these data should be interpreted with appropriate caution, as uncorrected values may overemphasize “noise” in the data interpretation. Future investigations with larger cohorts would improve the ability to detect significant associations and enable more robust correction procedures. Additionally, while predictive pathway analysis with PICRUSt helped us estimate the functional implications of alcohol-induced dysbiosis, these inferred functional outputs should be confirmed using shotgun metagenomics and direct functional assays in future work.

**Figure 5:**
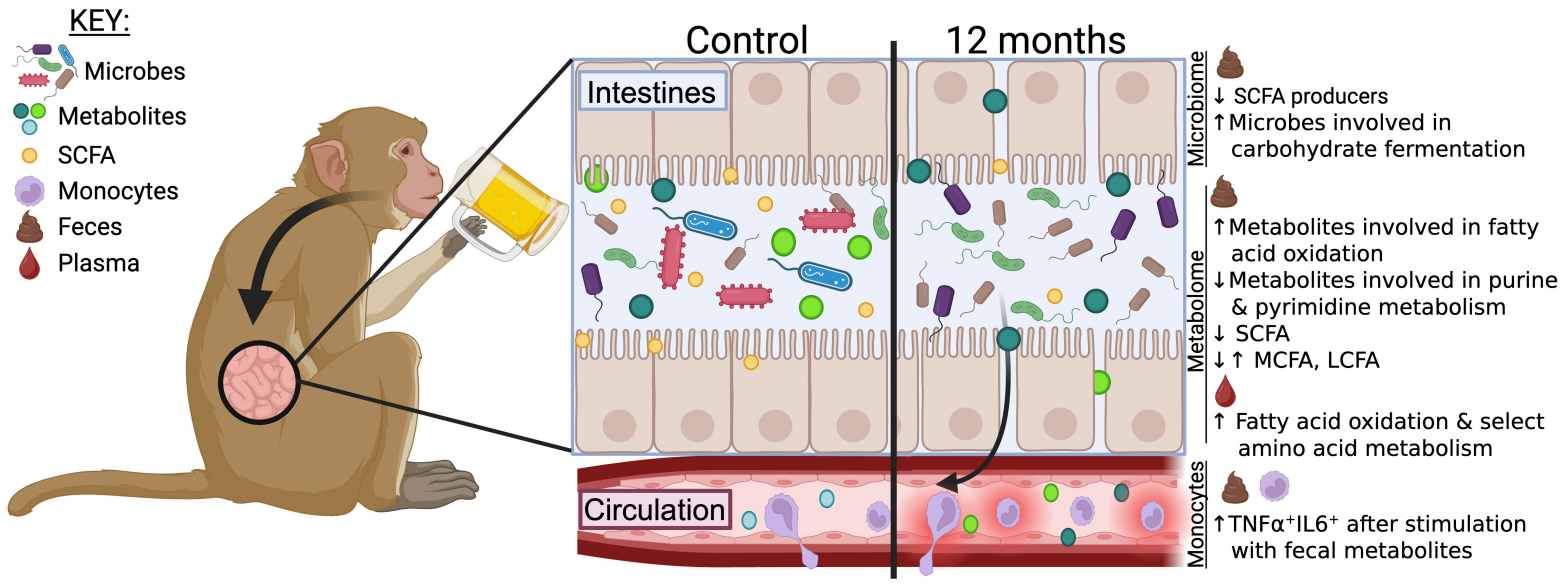
A graphical summary showing the results and the proposed mechanism of alcohol-induced dysregulation on the circulating immune system.

In conclusion, this study provides a comprehensive analysis of how alcohol disrupts the gut microbiome after chronic ethanol consumption but before the onset of liver damage in a macaque model of ethanol self-administration and its implications on the fecal and circulating metabolomes and monocyte function (Figure 5). Our data indicate that alcohol misuse skews the abundance of gut microbes without altering microbial diversity. We observed a reduced abundance of SCFA-producing bacteria and decreased levels of SCFA, likely contributing to compromised intestinal barrier integrity. Other changes were indicative of an inflammatory profile. These data enhance our understanding of how alcohol affects the circulating immune system, which could inform future research aimed at minimizing the immune consequences of alcohol consumption.

## Data Availability

The datasets presented in this study can be found in online repositories. The names of the repository/repositories and accession number(s) can be found at: https://www.ncbi.nlm.nih.gov/, PRJNA1276562.

## Supplementary material

Supplementary Figure S1 MaAsLin2 analysis reveals a *Bradymondales* genus to be negatively correlated to increasing ethanol consumption. Correlation plots of various bacterial abundances vs. average grams of ethanol intake per kg of the animal (EtOH, gkg). The false discovery rate (FDR) represents the significance of the correlation, the coefficient represents the slope of the correlation line, and *N* represents the number of samples evaluated.

Supplementary Figure S2 Twelve months of daily drinking induces beta-oxidation of fatty acids and reduces nucleoside metabolism. **(A)** sPLSDA of LC-MS analysis of macaque fecal samples from ethanol naive controls and after 12 months of daily drinking. **(B)** Volcano plot of differentially abundant metabolites in fecal samples after 12 months of alcohol use. **(C)** Bar plot depicting enrichment of differentially abundant metabolites in fecal samples after 12 months (terms with *p*-value < 0.1 and >2 genes). For the fecal LC-MS experiments: *n* = 9 controls and *n* = 8 after 12 months of drinking were used.

Supplementary Figure S3 Twelve months of alcohol use alters the levels of medium- and long-chain fatty acids in stool samples. Bar graphs showing the relative abundance of medium- and long-chain fatty acids present in stool samples before and after 12 months of alcohol use. Error bars were defined as ±standard error of the mean (SEM).

Supplementary Figure S4 Gating strategy. Gating strategy for trained immunity experiment.

Supplementary Table S1 Differentially abundant metabolites.
