# Supplementary figures and images for: Chronic alcohol consumption disrupts the gut microbial and metabolic landscapes
Source: Front Microbiol. 2026 May 13;17:1794794. doi: 10.3389/fmicb.2026.1794794 (PMC13212193; doi:10.3389/fmicb.2026.1794794)

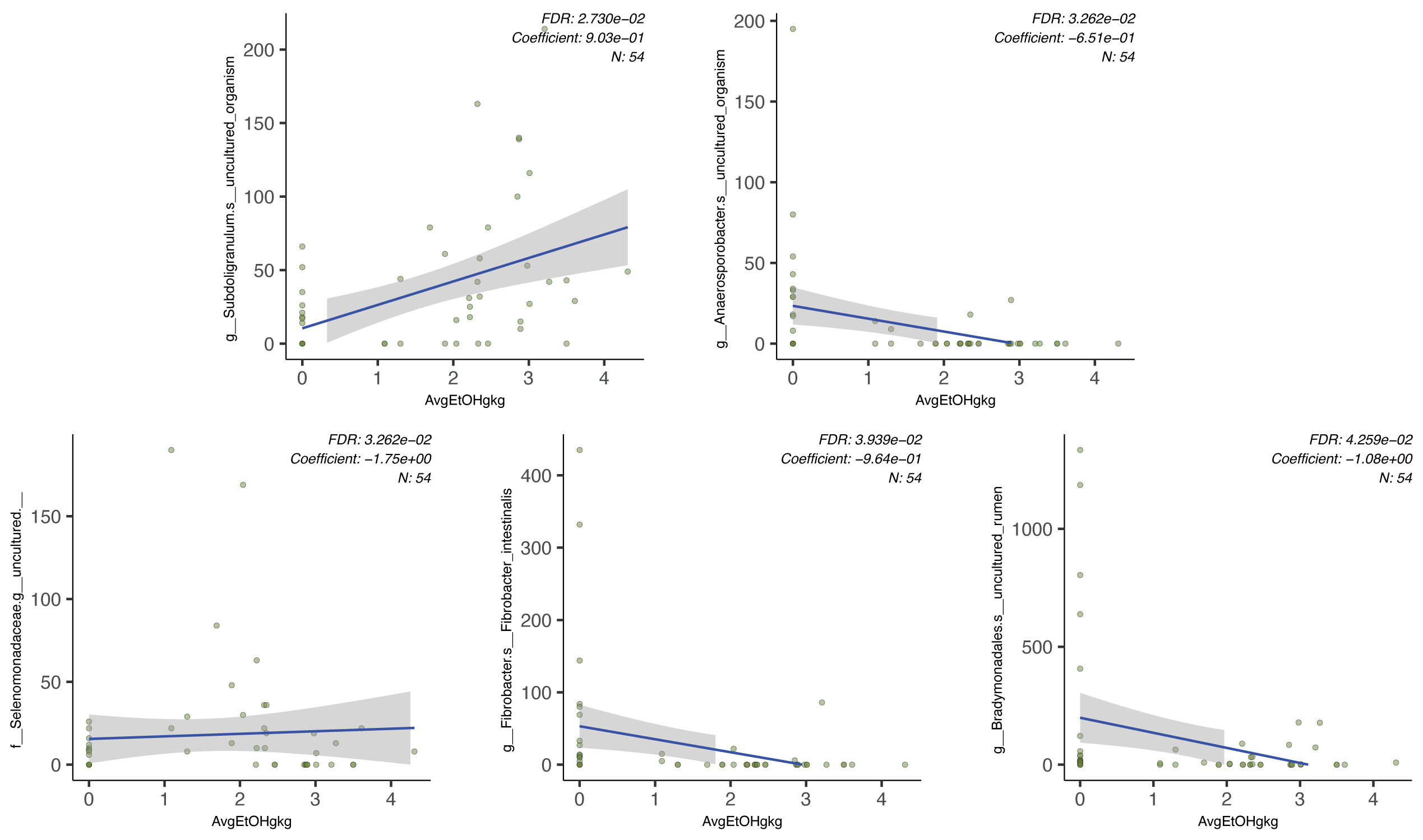

Supplement: Supplementary Figure S1 — MaAsLin2 analysis reveals a Bradymondales genus to be negatively correlated to increasing ethanol consumption. Correlation plots of various bacterial abundances vs. average grams of ethanol intake per kg of the animal (EtOH, gkg). The false discovery rate (FDR) represents the significance of the correlation, the coefficient represents the slope of the correlation line, and N represents the number of samples evaluated. [file Image_1.tif]

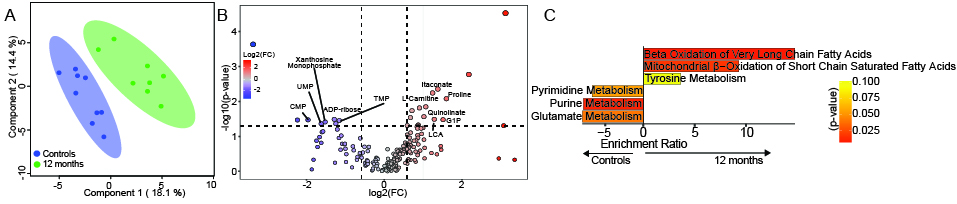

Supplement: Supplementary Figure S2 — Twelve months of daily drinking induces beta-oxidation of fatty acids and reduces nucleoside metabolism. (A) sPLSDA of LC-MS analysis of macaque fecal samples from ethanol naive controls and after 12 months of daily drinking. (B) Volcano plot of differentially abundant metabolites in fecal samples after 12 months of alcohol use. (C) Bar plot depicting enrichment of differentially abundant metabolites in fecal samples after 12 months (terms with p-value < 0.1 and >2 genes). For the fecal LC-MS experiments: n = 9 controls and n = 8 after 12 months of drinking were used. [file Image_2.jpeg]

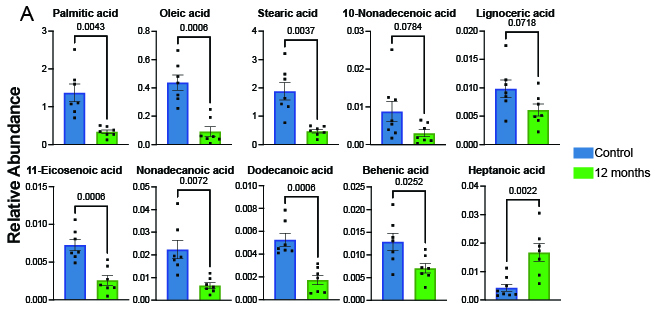

Supplement: Supplementary Figure S3 — Twelve months of alcohol use alters the levels of medium- and long-chain fatty acids in stool samples. Bar graphs showing the relative abundance of medium- and long-chain fatty acids present in stool samples before and after 12 months of alcohol use. Error bars were defined as ±standard error of the mean (SEM). [file Image_3.jpeg]

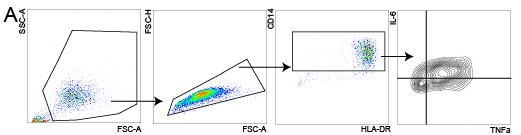

Supplement: Supplementary Figure S4 — Gating strategy. Gating strategy for trained immunity experiment. [file Image_4.jpeg]
